# Supplementary figures and images for: The influence of AHR on immune and tissue biology
Source: EMBO Mol Med. 2024 Sep 6;16(10):2290–8. doi: 10.1038/s44321-024-00135-w (PMC11473696; doi:10.1038/s44321-024-00135-w)

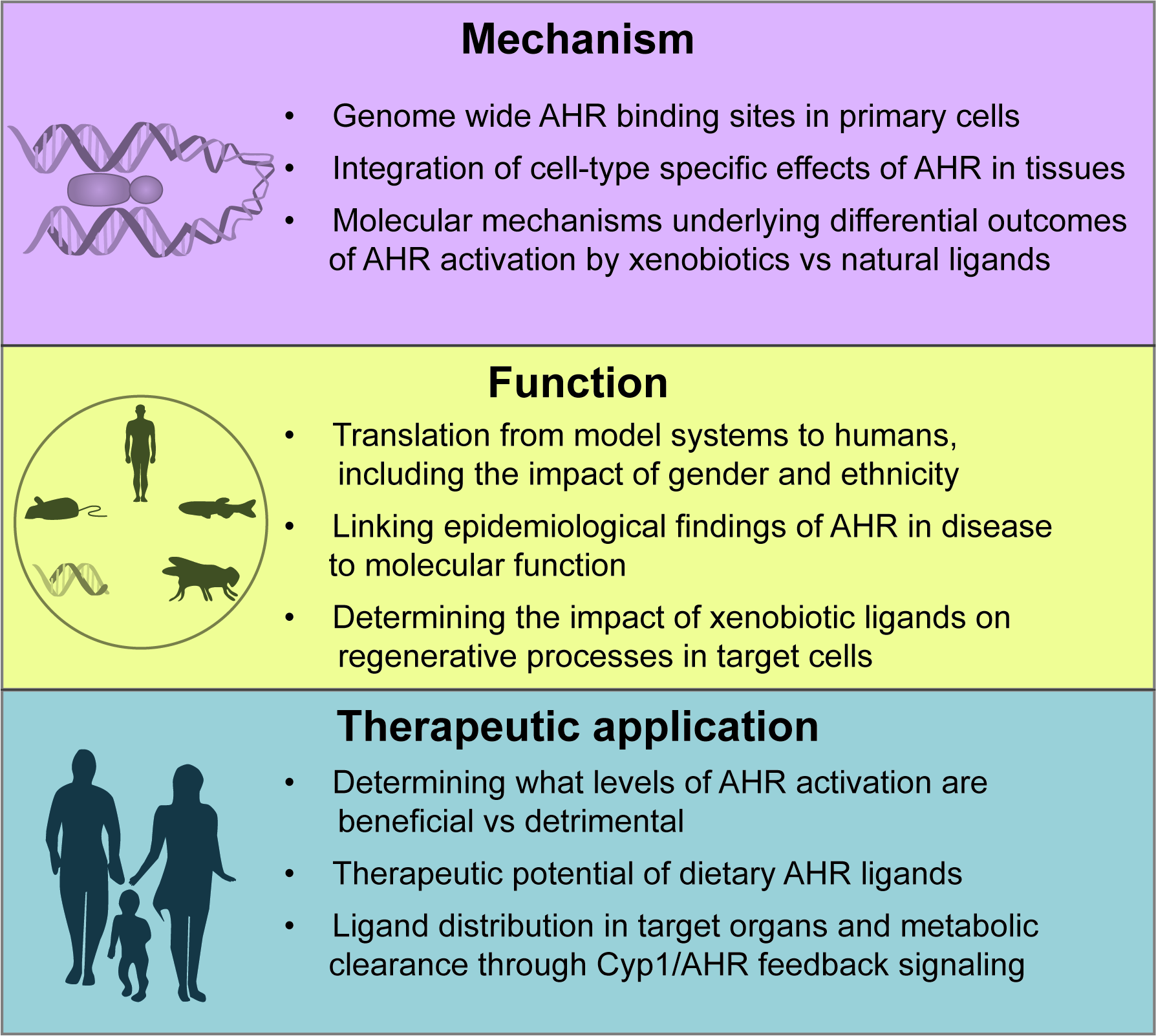

Supplement: Supplementary file 1 — Box 1 [file 44321_2024_135_MOESM1_ESM.tif]
